# Performance of Ultrasensitive Rapid Diagnostic Tests for Detecting Asymptomatic *Plasmodium falciparum*

**DOI:** 10.4269/ajtmh.19-0349

**Published:** 2019-12-09

**Authors:** Shunmay Yeung, David McGregor, Nicola James, Soy Ty Kheang, Saorin Kim, Nimol Khim, Po Ly, Siv Sovannaroth, Benoit Witkowski

**Affiliations:** 1 Clinical Research Department, Faculty of Infectious and Tropical Disease, Malaria Centre, London School of Hygiene and Tropical Medicine, London, United Kingdom;; 2 Health and Social Development (HSD), Phnom Penh, Cambodia;; 3 Institut Pasteur du Cambodge, Phnom Penh, Cambodia;; 4 National Center for Parasitology, Entomology and Malaria Control, Phnom Penh, Cambodia

## Abstract

Proposed interventions for eliminating drug-resistant *Plasmodium falciparum* malaria include the targeting of asymptomatic carriers through screening and treatment. We report on the diagnostic performance of the recently developed ultrasensitive rapid diagnostic test (uRDT) compared with screening with conventional RDTs (cRDT) and polymerase chain reaction (PCR) under field conditions in Cambodia in a total of 2,729 individuals. The *P. falciparum* positivity by quantitative PCR (qPCR) was 3.8% (26/678) in those screened during active case detection and 0.5% (10/2,051) in the cross-sectional survey. Compared with qPCR, the sensitivity of the uRDTs was 53.8% (95% CI: 33.4–73.4%) when used in active case detection and 60.0% (95% CI: 26.2–87.8%) in the cross-sectional survey. The uRDTs did not show a significant improvement in diagnostic performance over cRDTs when used for active case detection and for a malaria prevalence survey in the context of this low-transmission setting.

Like elsewhere in Southeast Asia, the burden of malaria in Cambodia has decreased dramatically over the last 15 years.^1,2^ However, the region is under the threat of *Plasmodium falciparum* resistance to artemisinin-based combination therapy.^3^ The Royal Government of Cambodia has committed to eliminating *P. falciparum* malaria by 2020 and all forms of malaria by 2025.^4^ Neighboring countries including Thailand, Vietnam, and Laos have also committed to malaria elimination.

To eliminate *P. falciparum* malaria, treatment strategies that solely focused on patients presenting with symptomatic malaria are unlikely to be sufficient. Asymptomatic carriage with low-level parasitaemias has now been well documented in the region and represents an important potential reservoir of infection.^5^

Until recently, efforts to screen for low-density infections have been limited by the available diagnostics. Conventional rapid diagnostic tests (cRDTs), although easy to use in the field and sufficiently sensitive for confirming infection in symptomatic infections, are not sensitive enough to detect low-level parasitaemias in asymptomatic infections. Conversely, molecular diagnosis using polymerase chain reaction (PCR) is highly sensitive but requires sophisticated laboratory facilities and, therefore, cannot be used operationally as a screening tool in remote forested areas where populations at a risk of malaria infection live.^6–8^

New “ultrasensitive rapid diagnostic tests” (uRDTs) have recently been developed and offer the exciting potential of a field-deployable highly sensitive test. Similar to cRDTs, they are based on the immunodetection of histidine-rich protein 2 (HRP2); however, they have a reported sensitivity of more than 50% with *P. falciparum* densities between 0.1 and 1 p/µL under laboratory conditions.^9^ Although promising, this new test needs to be evaluated under field conditions to determine its potential performance and utility in malaria elimination programs.

We aimed to evaluate the performance of uRDT in comparison with a cRDT and quantitative PCR (qPCR) under field conditions on the Cambodian–Thai border.

This study was nested within a large study of active case detection for malaria, which was carried out in Oddar Meanchey Province in Northwest Cambodia. In brief, in the 65 villages in the intervention arm, active case detection was carried out both reactively and proactively. Reactive case detection was carried out around “index cases,” that is, patients with symptomatic *P. falciparum* malaria who had presented to a village malaria worker or health center. Screening for malaria was carried out on members of their household, individuals who had traveled to the forest with them (“co-travelers”), and “high-risk” neighbors. High-risk individuals were defined as anyone reporting to have slept overnight in the forest in the past month and any individual with a reported fever in the previous 48 hours. In proactive case detection, all high-risk individuals in villages with high number of *P. falciparum* cases were screened proactively.

From June to December 2017, screening for malaria was carried out using three diagnostic tests: uRDT (Alere Malaria Ag P.f, Abbott, Lake Bluff, IL), cRDT under routine use (Standard Diagnostics, Bioline, Gyeonggi-do, Republic of Korea) with HRP-II and pLDH antigen RDT (Alere Malaria Ag P.f, Abbott), and nested PCR on blood spot samples conducted at the Institute Pasteur laboratory in Phnom Penh, as previously described.^10^ In addition, a cross-sectional survey was conducted from November to December 2017 in 17 of the highest burden villages. All participants found positive for malaria by any diagnostic tests were offered treatment with the first-line artemisinin-based combination therapy.

Both village malaria workers and survey data collectors were provided with training on how to take blood samples and carry out RDTs correctly as well as preparing filter paper and preprepared 96-well plates for subsequent PCR analysis. Nucleic material extraction from dried blood spot and screening for any malaria species infection and then specific *P. falciparum* infection were carried out according to the methodology of Canier et al.^11^ Results of RDTs were recorded on paper-based forms by field workers, uploaded into an Excel (Microsoft Corporation, Redmond, WA) database, and then merged with the results of qPCR analysis using the unique participant identification number. The descriptive statistical analysis was carried out using Stata 15.1 (StataCorp, College Station, TX). Using qPCR as the reference standard, sensitivity and specificity were calculated. As the main target population for malaria control and elimination in this region is forest goers, a subgroup analysis was carried out on this group, defined as anyone who had reportedly slept overnight in the forest in the previous month.

Ethical approval for this study was obtained from the Cambodian National Ethical Committee for Human Research and the London School of Hygiene & Tropical Medicine Ethics Committee. Participants were included in the study only if informed consent was obtained (parents or guardians provided consent for children younger than 18 years). Children older than 12 years were also required to provide oral assent for participation in addition to the consent of their parent or guardian.

In total, 2,729 individuals were tested using all three diagnostic tests: 678 during active case detection and 2,051 during the cross-sectional survey. The positivity rate for *P. falciparum* infections by qPCR analysis was 3.8% (26/678) and 0.5% (10/2,051) for active case detection and the cross-sectional survey, respectively. The cRDT and uRDT results were compared with the qPCR results as the reference standard (Table 1). When used for active case detection, the sensitivity of the cRDT and uRDT for *P. falciparum* malaria was 46.2% (95% CI: 26.6–66.6%) and 53.8% (95% CI: 33.4–73.4%) and specificity was 98.2% (95% CI: 96.8–99.0%) and 96.8% (95% CI: 95.1–98.0%), respectively (*P* = 0.0127). Ten samples were positive by qPCR and negative by both types of RDTs and 11 samples were negative by qPCR but positive by both types of RDTs. When used in the cross-sectional survey, the sensitivity for *P. falciparum* was 60.0% (95% CI: 26.2–87.8%) and specificity was 99.8% (95% CI: 99.5–99.9%) for both cRDT and uRDTs compared with the reference PCR (*P* = 1). Four samples tested positive by qPCR and negative by both RDTs and vice versa. Figure 1 shows the parasite densities by the RDT result. Sub-analysis conducted on just forest goers showed similar findings (results not shown).

**Table 1:** Comparison between PCR and cRDTs and uRDTs for screening for *Plasmodium falciparum* during active case detection and the cross-sectional survey

	cRDT+	cRDT−	uRDT+	uRDT−
Active case detection				
Total	24	654	35	643
PCR Pf+	12	14	14	12
PCR Pf−	12	640	21	631
Sensitivity (%)	46.2 (95% CI: 26.6–66.6%)		53.8 (95% CI: 33.4–73.4%)	
Specificity (%)	98.2 (95% CI: 96.8–99.0%)		96.8 (95% CI: 95.1–98.0%)	
Cross-sectional survey				
Total	10	2,041	10	2,041
PCR Pf+	6	4	6	4
PCR Pf−	4	2,037	4	2,037
Sensitivity (%)	60.0 (95% CI: 26.2–87.8%)		60.0 (95% CI: 26.2–87.8%)	
Specificity (%)	99.8 (95% CI: 99.5–99.9%)		99.8 (95% CI: 99.5–99.9%)	

cRDT = conventional rapid diagnostic test; PCR = polymerase chain reaction; Pf = *Plasmodium falciparum*; uRDT = ultrasensitive rapid diagnostic test.

**Figure 1.**
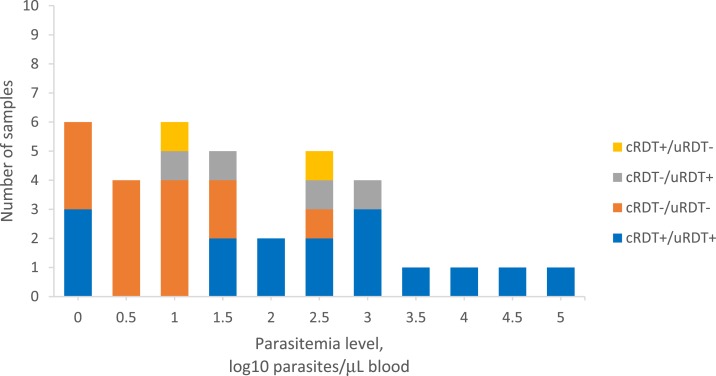
Distribution of estimated parasite density calculated from polymerase chain reaction cycle threshold levels, in comparison with conventional rapid diagnostic tests (RDTs) and ultrasensitive RDTs (uRDTs) for screening for *Plasmodium falciparum* during active case detection and cross-sectional survey in Cambodia.

To our knowledge, this is the first published study to report on the diagnostic performance of uRDTs under field conditions. The uRDTs were found to have similar sensitivity and specificity to both cRDTs when used in active case detection and in a cross-sectional survey. This differs from what has been reported from laboratory-based studies. Das et al.^9^ reported a uRDT sensitivity of 44% (95% CI: 15–77%) and specificity of 99.8% (95% CI: 99–100%) compared with quantitative reverse transcriptase PCR (qRT-PCR) and compared with a sensitivity of 0% (95% CI: 0–37%) and a specificity of 100% (95% CI: 99–100%) for the cRDT (Bioline, Standard Diagnostics) in whole blood specimens collected from asymptomatic individuals in a cross-sectional survey in Myanmar, where the positivity was 1.8% (9 of 493) by qRT-PCR. An analysis of 247 blood samples from a cross-sectional survey in Papua New Guinea by Hofmann et al.^12^ reported a uRDT (Malaria Ag P.f Ultra-Sensitive, Standard Diagnostics) sensitivity of 27% compared with 15% for cRDTs (Malaria Ag P.f/P.v, Standard Diagnostics) with qPCR used as a reference method. It should be noted that the PCR techniques used differed between studies and may explain some of the differences in the results.

There are a number of reasons why the uRDTs did not show enhanced sensitivity compared with cRDTs in this study. The uRDTs were stored in an air-conditioned office; however, it is possible that for brief periods during power cuts and transportation, they may have been exposed to temperatures of up to 35°C—higher than the recommended maximum of 30°C. Incorrect use by the field workers is also possible but very unlikely, as they received extensive training, were closely supervised and worked in pairs so that two people checked the results. A final consideration is the possibility of HRP2 deletions; however, this has not yet been reported in the region.

Despite their reported enhanced sensitivity, uRDTs did not show a significant improvement in diagnostic performance over cRDTs when used for active case detection and for a malaria prevalence survey in the context of this low-transmission setting. Further field evaluations for uRDTs are required in different epidemiological and under different operational settings to establish where they are likely to be of most benefit.

## References

[1] Sochantha T Hewitt S Nguon C Okell L Alexander N Yeung S Vannara H Rowland M Socheat D, 2006 Insecticide-treated bednets for the prevention of *Plasmodium falciparum* malaria in Cambodia: a cluster-randomized trial. Trop Med Int Health 11: 1166–1177. 16903880 10.1111/j.1365-3156.2006.01673.x

[2] WHO, 2018 World Malaria Report 2018. Geneva, Switzerland: World Health Organization.

[3] Ashley EA Tracking Resistance to Artemisinin Collaboration, 2014 Spread of artemisinin resistance in *Plasmodium falciparum* malaria. N Engl J Med 371: 411–423. 25075834 10.1056/NEJMoa1314981 PMC4143591

[4] Ministry of Health Kingdom of Cambodia, 2017 Cambodia Malaria Elimination Action Framework (2016–2020) | MESA. Available at: http://mesamalaria.org/resource-hub/cambodia-malaria-elimination-action-framework-2016-2020. Accessed October 1, 2019.

[5] Bousema T Okell L Felger I Drakeley C, 2014 Asymptomatic malaria infections: detectability, transmissibility and public health relevance. Nat Rev Microbiol 12: 833–840. 25329408 10.1038/nrmicro3364

[6] Gryseels C 2015 High mobility and low use of malaria preventive measures among the Jarai male youth along the Cambodia-Vietnam border. Am J Trop Med Hyg 93: 810–818. 26283747 10.4269/ajtmh.15-0259 PMC4596604

[7] Guyant P Canavati SE Chea N Ly P Whittaker MA Roca-Feltrer A Yeung S, 2015 Malaria and the mobile and migrant population in Cambodia: a population movement framework to inform strategies for malaria control and elimination. Malar J 14: 252. 26088924 10.1186/s12936-015-0773-5 PMC4474346

[8] Bannister-Tyrrell M 2019 Forest goers and multidrug-resistant malaria in Cambodia: an ethnographic study. Am J Trop Med Hyg 100: 1170–1178. 30860021 10.4269/ajtmh.18-0662 PMC6493920

[9] Das S 2017 Performance of a high-sensitivity rapid diagnostic test for *Plasmodium falciparum* malaria in asymptomatic individuals from Uganda and Myanmar and naive human challenge infections. Am J Trop Med Hyg 97: 1540–1550. 28820709 10.4269/ajtmh.17-0245 PMC5817764

[10] Hoyer S 2012 Focused screening and treatment (FSAT): a PCR-based strategy to detect malaria parasite carriers and contain drug resistant *P. falciparum*, Pailin, Cambodia. PLoS One 7: e45797. 23049687 10.1371/journal.pone.0045797 PMC3462177

[11] Canier L 2015 Malaria PCR detection in Cambodian low-transmission settings: dried blood spots versus venous blood samples. Am J Trop Med Hyg 92: 573–577. 25561570 10.4269/ajtmh.14-0614 PMC4350552

[12] Hofmann NE 2018 Assessment of ultra-sensitive malaria diagnosis versus standard molecular diagnostics for malaria elimination: an in-depth molecular community cross-sectional study. Lancet Infect Dis 10: 1108–1116. 10.1016/S1473-3099(18)30411-0 30170986

